# Advances in construction and modeling of functional neural circuits *in vitro*

**DOI:** 10.1007/s11064-022-03682-1

**Published:** 2022-08-09

**Authors:** Siu Yu A. Chow, Huaruo Hu, Tatsuya Osaki, Timothée Levi, Yoshiho Ikeuchi

**Affiliations:** 1 Institute of Industrial Science, The University of Tokyo, Tokyo, Japan; 2 Department of Chemistry and Biotechnology, School of Engineering, The University of Tokyo, Tokyo, Japan; 3 Institute for AI and Beyond, The University of Tokyo, Tokyo, Japan; 4 IMS laboratory, CNRS UMR 5218, University of Bordeaux, Talence, France

## Abstract

Over the years, techniques have been developed to culture and assemble neurons, which brought us closer to creating neuronal circuits that functionally and structurally mimic parts of the brain. Starting with primary culture of neurons, preparations of neuronal culture have advanced substantially. Development of stem cell research and brain organoids has opened a new path for generating three-dimensional human neural circuits. Along with the progress in biology, engineering technologies advanced and paved the way for construction of neural circuit structures. In this article, we overview research progress and discuss perspective of *in vitro* neural circuits and their ability and potential to acquire functions. Construction of *in vitro* neural circuits with complex higher-order functions would be achieved by converging development in diverse major disciplines including neuroscience, stem cell biology, tissue engineering, electrical engineering and computer science.

## Introduction

During development *in vivo*, cells can flawlessly generate the whole human body. One of the highlights of embryogenesis is the formation of the brain, which is achieved by the most complex choreography and orchestration of cells. In addition to cellular processes that occur during formation of other organs (e.g. proliferation, differentiation, and migration), diverse types of neurons extend and connect their dendrites and axons, and go through maturation process, to generate complex networks of neural circuits. The functions of neurons as the key elements of the brain are to propagate action potential within the cells and to transmit and receive activity through synapses. Neurons can be cultured *in vitro*, go through maturation process, retain their elementary functions as neurons, and exhibit characteristics of diverse cell types. Therefore, it would be natural to hope and be optimistic for the formation of functional neural circuits *in vitro*. However, construction of functional neural circuits *in vitro* could be considered as one of the most difficult tasks that have been sought after for a long time.

Functions of neural circuits could be appreciated from two perspectives. Considering neurons as elements for neural circuits, abilities or characteristics of neurons emerged by forming networks could be considered as functions of the circuits. Considering neural circuits as sub-systems of the brains, activity or response that provide mechanistic processes for the brain performance could be functions of the circuits as well. With these criteria, *in vitro* neural circuits have demonstrated their diverse mechanistic functions including complex oscillation activity and network plasticity. It is fair to assume that more unique and higher-order functions could be achieved by constructing *in vitro* neural circuits with certain structures that are organized to acquire signal processing capability. Ultimately, neural circuits with desired functions might be generated by connecting neurons just as a computer assembled with semiconductor and other elements.

Attempts to construct neural circuits with certain functions directly link with efforts to understand minimal structural requirements of the circuits which execute mechanistic functions within the brain. If a particular function of the brain is executed by a specific neural circuit, in theory, the same function would be reproduced by an *in vitro* neural circuit that precisely mimics the functional *in vivo* circuit structure. The neural circuit structures in the brain have been explored in detail by classical anatomical approaches and recent connectome studies [1, 2]. The functions of the neural circuits *in vivo* have been revealed rapidly by various approaches including electrophysiology, fMRI, EEG, electrode array, calcium imaging and optogenetics [3–6]. As structures and functions of the brain circuits are being clarified, the need for *in vitro* neural circuit models that can investigate the circuits’ fundamental mechanisms increased correspondingly.

Neurological diseases indicated that disruption of various circuits triggers diverse functional defects of the brain. It is essential to create *in vitro* disease models that precisely recapitulate the symptomatic regions or circuits of the brain to understand their pathophysiological mechanisms. Correspondingly, *in vitro* disease modeling provide important insights into functionality of circuits by understanding their deregulation phenotypes in relation to the disease symptoms.

Conventional two-dimensional culture of neurons generate limited functionality due to random orientation and interaction of neurons. Efforts have been made to mimic neural circuits with neurons cultured *in vitro* to provide structural basis for functionalization of neurons. Various engineering techniques have been developed to control positioning, connection, and assembly of living neural cells that are fragile, unstable and hard to control. In parallel, stem cell research enabled formation of three-dimensional brain-like tissues (brain organoids) from human stem cells, which mimic brain structures including layers of subtypes of neurons. Brain organoids showed great potentials of creating neural circuit models that resembles the brain structure and functionality more than two-dimensional cell cultures. Research of neural circuit formation is currently dynamically changing and advancing through convergence of engineering and biology and incorporation of other diverse research fields. Could higher-order functions emerge and be recognized in the *in vitro* neural circuits by extending the current efforts to control and assemble neural cells? We review the progress and perspectives toward building functional neural circuits in this review.

**Fig. 1:**
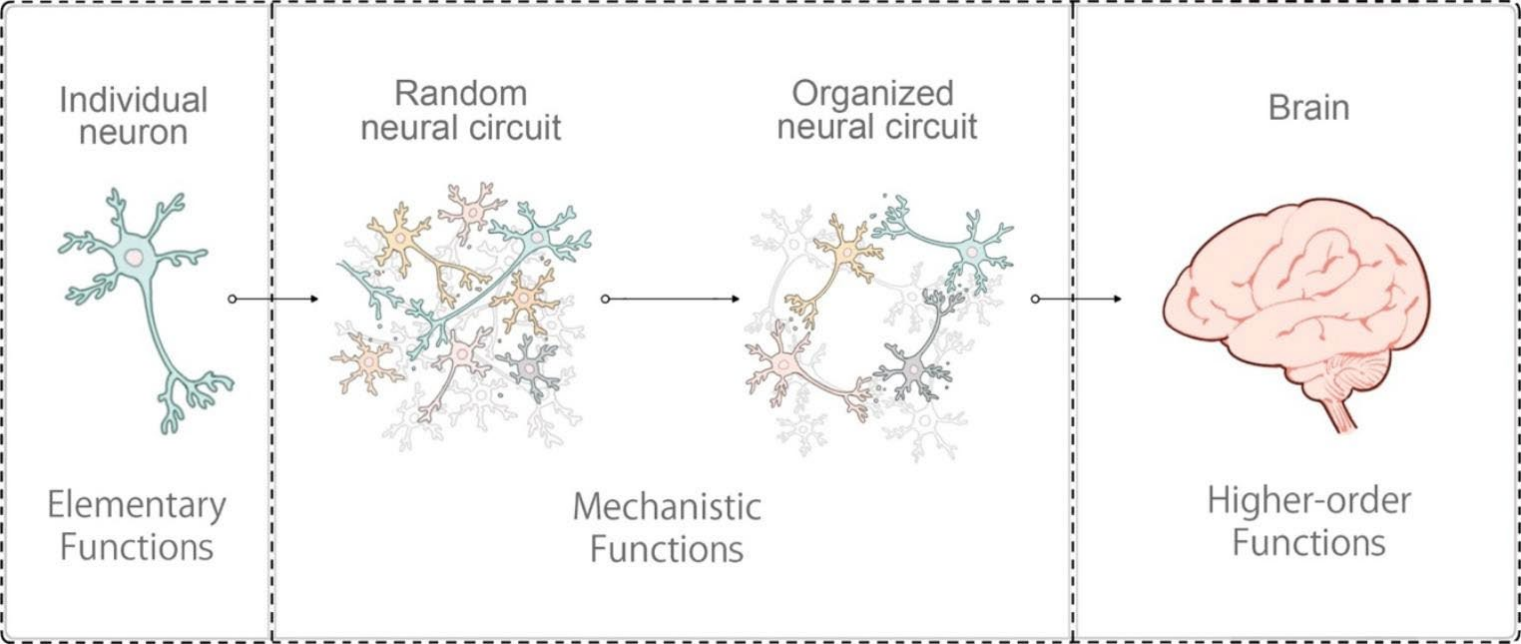
Levels of functionality and organization of neurons

Individual neurons exhibit their “elementary functions” including generation of action potentials (left). The neurons form circuits and gain “mechanistic functions” including generation of complex network activity (middle). The brain exhibit “higher-order functions” by coordinating activity of the neural circuits (right).

## Culture of neurons

### Primary neuron culture

The foundation for the construction of a functional neural circuit *in vitro* relies on the culture of neurons capable of performing their elementary functions (Fig. 1). Dissociated neuronal culture of rat neurons was first developed over 40 years ago [7]. Isolated primary rodent neurons can develop and acquire morphologies and characteristics resembling neurons *in vivo*. Diverse types of neurons from different regions of the brain can be cultured [8–12]. The cultured neurons mature *in vitro* and exhibit ability to form synapses and induce action potentials. Over the years, primary rodent neuronal cultures have been widely applied as a model system and significantly facilitated our understanding of neuron physiology and functions, particularly in the field of morphogenesis and synaptogenesis. Defined media and growth factors have been established for the primary neuron cultures [13, 14], which paved the way for further development of cell and tissue culture methods. Importantly, the *in vitro* cultured neurons can produce periodic synchronized burst activity [15], which could be considered as a relatively simple yet important network function, and has been proposed to model sleep/wake cycles [16, 17].

### Stem cells

Primary culture of rodent neural stem cells (NSCs) have facilitated understanding of their asymmetric/symmetric division and differentiation *in vitro* [18]. Culture of NSCs as free-floating aggregates, known as neurospheres, was developed as an efficient method to maintain and expand NSCs [19, 20], illustrating the importance of cell-cell interaction for the growth of NSCs. Primary cultured neurons must be isolated from animals for each experiment. Also, primary NSCs cannot proliferate infinitely. Therefore, cell lines capable of differentiating into neurons have been used as alternative cell models, such as Neuro2A (mouse line) and SH-SY5Y (human line) [21–23]. While these cell lines present the advantage of permanent proliferation, they have insufficient differentiation potential to produce mature neurons. Although human primary NSCs have also been cultured [24, 25], difficulty in obtaining human specimens highlights the need for alternative methods to acquire human neurons.

Embryonic stem (ES) cells isolated from the inner cell mass of fertilized eggs can be differentiated into diverse cell types including neurons [26–32]. In 1998, human ES cell lines were generated and provided a major advance in the investigation of human neurons [33–36]. However, ethical debates restricted the usage of ES cells derived from human embryos. The research of human neurons was then boosted by the revolutionary invention of human induced pluripotent stem cells (iPS cells), in which somatic cells are reprogrammed to acquire pluripotency. Somatic cells could become pluripotent stem cells by overexpression of only four transcription factors (OCT3/4, KLF4, SOX2, c-MYC). Mouse iPS cells were first reported in 2006, followed by human iPS cells in 2007 [37, 38]. Human iPS cells can be produced from any individual, which is ideal for personalized regenerative medicines, disease models, and drug development. Methods for differentiating iPS cells into various neural cells have been established [39–42]. In parallel, methods to directly reprogram somatic cells into induced neurons (iNs) have also been developed and utilized widely [43]. These reliable and powerful culture methods provided access to human neurons *in vitro* for cellular and molecular neuroscience and other fields of study.

### Slice culture

While the above-mentioned neurons are randomly oriented in the culture, *in vivo* neural circuits are complex yet well organized. To extract and obtain the functional neural circuits, acute slices of the brain from animals are prepared. Acute slices of rodent hippocampus are the most studied *ex vivo* neural circuits and have significantly advanced the study of synaptic plasticity [44–47]. Brain slices of multiple regions (e.g., cortex and thalamus) have also been developed, thereby providing researchers opportunities to investigate both local microcircuits and the communication between distant target regions [48–50]. They can exhibit oscillatory activity similar to the neural circuits *in vivo* [47, 51], indicating that the neuronal circuits are at least partially functional in the slices. Organotypic brain slices can be cultured and maintained *ex vivo* for a couple of weeks, which allows researchers to observe temporal (developmental) changes of the cells. However, it is challenging to keep the circuit structure perfect for an extended period of culture, since the cells tend to migrate within and out from the tissue. Furthermore, a major limitation of brain slice methods is that it is not applicable to humans, which restricts the potential for drug development and human brain studies.

### Engineered circuits in two-dimensional culture of neurons

The cultured neurons have the property of spontaneously extending neurites and forming synapses with other neurons. If no physical restriction is applied to the neurites, they will extend in random directions, form synapses between accidentally encountered neurons, and create circuits without architectural plans. Synapses can even be formed between the dendrites and axons extended from the same cell [52].

Towards establishing organized neural circuits in two-dimensional culture, spatial control of neurons and their processes is one of the most critical techniques. Since the Campenot chamber was first introduced in 1977, various microfluidic culture chips have been developed and utilized to control neurites. Typically, these devices allow separation between cell bodies and axons through control of neurite outgrowth by size. Microslits in a wall separating multiple culture chambers are designed to be wide enough for axons (approximately 0.5 μm in width) to pass through, whereas cell bodies (several tens of µm wide) cannot pass through. Thus, neurons plated into one chamber stay inside of the chamber, but axons grown from the neurons pass through the microslits and extend into another chamber. While porous membranes can be also used for separation of axons from cell bodies, microslit chips are superior for more elaborate designs aimed at circuit formation. By using the devices, neurons can be connected via axons within the microgroove to create circuit structures. Furthermore, more advanced microfluidic devices have been applied not only for connecting two types of neurons but also for connecting more than three types of neurons to achieve co-culture of multiple cell types [53]. Techniques have been developed to control the direction of synaptic connections by devising the shape of microgrooves and compartmentalized chambers. For example, by arranging a short microslit for dendrites to pass through on one side, and a long microslit for axons on the other side, the directionality of synapses can be modeled [54]. Also, other microfluidic studies demonstrated the control of axonal outgrowth with asymmetric channels and gradient generator (e.g. Netrin-1 gradient) to regulate axonal outgrowth direction [55, 56].

It is also possible to direct neuron outgrowth by micropatterning and microstructures on the culture surface [57–61]. Application of technologies that dynamically change the patterns on the culture surface by light or heat have been invented as well [62–65]. Additionally, more advanced axon-controlling methods such as microrobot and microplates have been proposed [66–68]. Advancements in technologies to control neurons will allow us to design and create neural circuits.

Over the years, various technologies have been developed one after another in two-dimensional neuron cultures, and related ongoing research is steadily progressing towards the establishment of neural circuits with organized structures. The development of these circuits has made it possible to study neural plasticity and generate simple oscillatory activities *in vitro*, but improvements are still required to generate more complicated activities and acquire higher-order functions.

## Three-dimensional culture of neurons

### Assembly of three-dimensional neuronal tissues

**Fig. 2:**
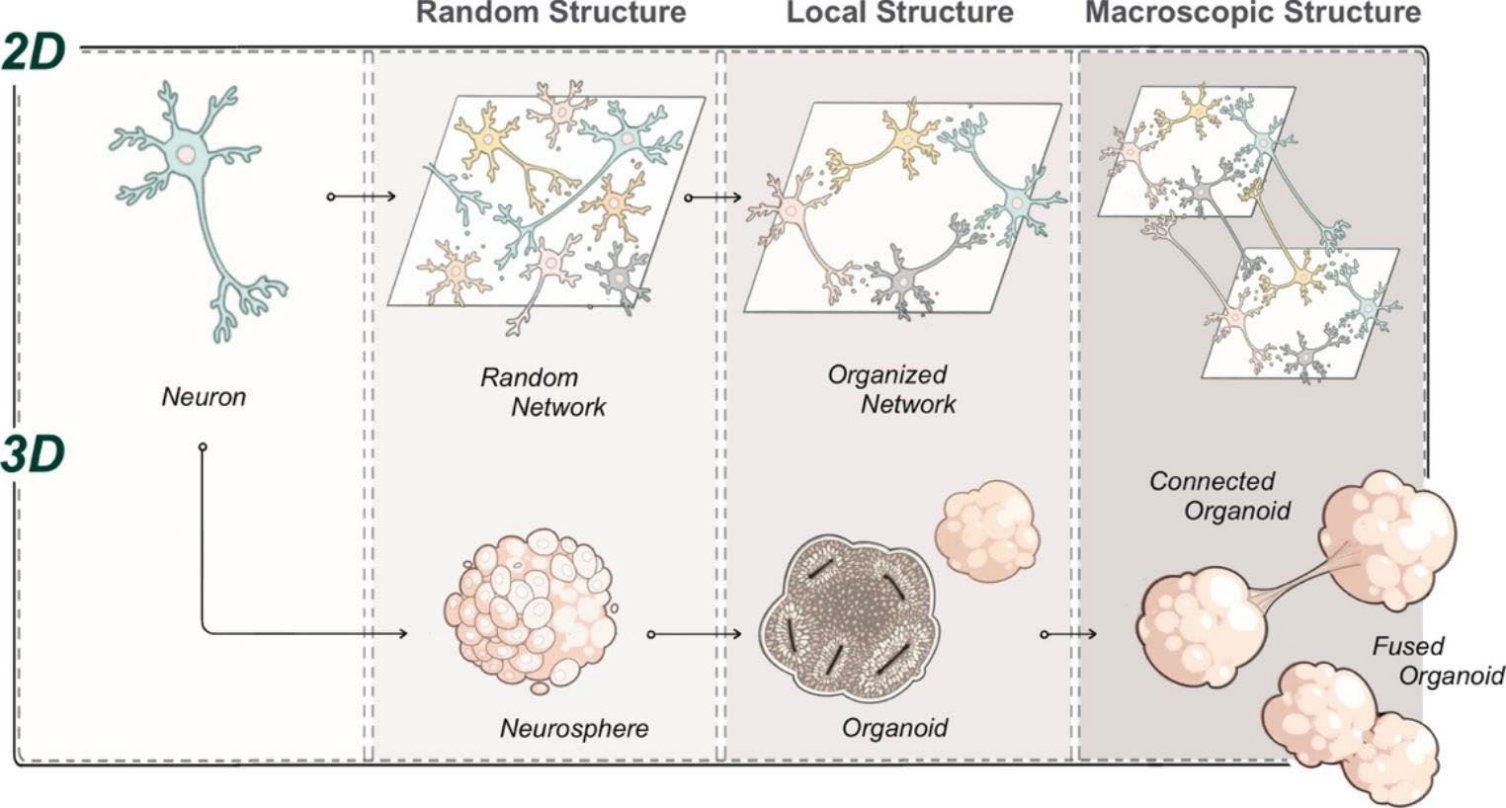
Development of *in vitro* neuronal cultures with advanced structures

Two-dimensional cultures of neurons contributed tremendously to the understanding of neuronal physiology, taking advantage of their accessibility for observation and ease of manipulation. Neuron culture systems and engineering techniques in three dimensions could advance the modeling of more complex neural circuits (Fig. 2).

As mentioned earlier, primary NSCs are often cultured in three-dimensional as neurospheres. The three-dimensional environment facilitates cell-cell interactions within the tissues and exposure to extracellular matrix produced by themselves, which mimic the *in vivo* environment and promote cellular maturation. More importantly, neurons could form synapses and generate unorganized circuits within the tissues, although the internal structure of neurospheres do not necessarily mimic the brain.

Inter-regional connections are critical for coordination and information processing of the brain. To achieve this, methods to integrate the neurospheres as building blocks have been developed [69]. Neural networks were built with three-dimensional neuronal tissues mutually connected by axons [70]. Importantly, these studies demonstrated that neurons could extend their axons out from the tissues, with their cell bodies remaining in the tissue even in the *in vitro* culture. The physical segregation between axons and cell bodies is naturally achieved *in vivo*, but often cells migrate out of tissues *in vitro*, impeding the creation of neural circuits. This indicates that three-dimensional assembly of cells within the neurospheres provides strong adhesion between cells, serving as a robust structural foundation to build circuit-like structures.

### Brain organoids

Stem cell research independently propelled three-dimensional tissue culture technologies. Dr. Yoshiki Sasai’s group first demonstrated that cultured three-dimensional embryoid bodies of mouse ES cells can spontaneously form neural tube-like neuroepithelial structures with inner lumens [71]. The neuroepithelia can differentiate and form cortical layer structures, recapitulating early *in vivo* cortical embryogenesis, which was not typically observed in the neurospheres that are mentioned in the previous section. This marked an important turning point for *in vitro* neuroscience research by demonstrating that stem cells can self-organize to produce complex and organized three-dimensional tissues.

Similarly, human iPS cells and ES cells in three-dimensional aggregates can undergo differentiation and self-organization that result in tissues with brain-like structures [72]; such brain-like tissues are called human brain organoids. The development of brain organoids opened new opportunities to model human brain development *in vitro*. In addition, brain organoids derived from patient iPS cells or genetically engineered disease-model human stem cells can enhance our understanding of disease pathogenesis and facilitate the search for new drugs.

What distinguishes brain organoids from previous human neuron models is the structures of cellular assemblies e.g. spontaneously organized layers, similar to the *in vivo* human brain development. This could be extremely beneficial in the establishment of complex neural circuit constructs. Also, a population of neural stem cells named outer radial glia (oRG) cells that are present in human brains has been identified in the organoids, indicating that organoids are good models of the human brain.

Various regions of our brain are mostly originated from a neural tube, in which each region gradually segments and acquires its identity. Formation of the brain regions are controlled by morphogens that are differently expressed along the anterior-posterior and dorso-ventral axes of the neural tube and the body. Organoids resembling different brain regions can be created by mimicking and reproducing the corresponding developmental processes through treatment with extrinsic factors [73]. Brain organoid has evolved as an excellent technology that successfully simulates the early stages of brain development and generates region-specific neuron subtypes.

Strikingly, organoids cultured for long periods of time (several months) have been reported to exhibit complex oscillatory activities, with the activity patterns similar to those of premature infants’ brainwaves [74]. Brain organoids cultured for over a year have shown signs of post-natal stages between 250 and 300 days following culture [75]. In addition, from the perspective of neural circuit formation, complex macroscopic circuits generation is challenging as interregional circuit cannot form spontaneously or randomly within conventional organoids despite after extended duration of culture.

Conventional two-dimensional culture of neurons form random networks in a dish. Bioengineering techniques to generate organized networks mimicking local circuit structures have been developed. This could be further incorporated into a network of networks that mimic macroscopic circuit structures. Neurons could be also cultured in a three-dimensional aggregate known as a neurosphere. The brain organoids bearing advanced three-dimensional internal structures can be generated by spontaneous differentiation and maturation of stem cells in a spheroid. The brain organoids could be further used to develop advanced structures including fused and connected organoids.

## Brain organoids as disease models

### Developmental brain diseases

In the past, disease pathogenesis of the human brain was largely explored through post-mortem patient biopsy, which provides only snapshots of the disease. Among various types of disease models that allow researchers to actively investigate disease pathogenesis and develop treatment, brain organoids demonstrate their unique characteristics of mimicking the human brain.

In 2013, Lancaster et al. reported the differentiation of cerebral organoids from iPS cells derived from microcephaly patients, marking the first time in which brain organoids were used to model disease phenotypes [72]. The patient-derived cerebral organoids were smaller in size and displayed premature neural differentiation than non-patient cerebral organoids. Brain organoids also contain a diverse cellular composition. By understanding the cellular heterogeneity of organoids, cell-type specific phenotypes of neurological disorders can be uncovered. For example, a recent paper modeled the neurodevelopmental phenotypes of tuberous sclerosis (TSC) [76]. Single-cell RNA-seq analyses identified a sub-group of progenitor cells in TSC patient-derived organoids that could give rise to both cortical tubers and tumors.

The unique ability of brain organoids to self-organize and mature to form layered structures led early studies using patient-derived brain organoids to reveal critical physical phenotypes *in vitro*. As our brain’s higher-order capacity depends on the functionality of neural circuits and their coordination, most neurological diseases are not limited to structural and cellular alterations of the brain, but also functional changes at a network level. As emerging evidence indicates that brain organoids contain intrinsically complex networks, the potential of brain organoids to study network disruptions in disease states has come under the spotlight. Sun *et al*. modeled Angelman Syndrome, a neurodevelopmental disease characterized by developmental delays and seizures, with CRISPR-mediated UBE3A knock-out (KO) human ES cells [77]. The KO organoids exhibited seizure-like hyperactivity and synchronous network activity that was reversed by the big potassium channel blocker paxilline. Paulsen et al. looked at risk genes involved in autism spectrum disorder (ASD), including SUV420H1. SUV420H1 mutant organoids displayed reduced network burst frequency and duration [78]. Correspondingly, the SUV420H1 mutant organoids exhibited disruption of developmental cell type specification. More advanced organoid disease models were established for Timothy Syndrome (TS). Hypersynchronous activity was observed in TS patient-derived dorsal forebrain organoids, which was more prominent when fused with ventral forebrain organoids (a.k.a. assembloids; Please see below "Multi-regional organoids" section) [79]. Another assembloid model for Rett Syndrome exhibited hypersynchrony, recurring epileptiform-appearing spikes and high frequency oscillations, despite no obvious deficit in cytoarchitecture [80]. Gamma oscillation in mutant assembloid was partially rescued by the administration of Pifithrin-α, a p53 inhibitor recently shown to be able to rescue premature aging induced by MeCP2 mutation, highlighting the potential of brain organoids in drug testing [80].

### Neurodegenerative diseases and neurological damages

Other than neurological diseases that happen early in development, brain organoids have also shown promise in the study of late-onset, neurodegenerative diseases (NDDs). These diseases uniquely affect specific regions of the nervous system, but have the common hallmark of aggregated protein accumulation, linked to neuronal damage and their loss. NDDs cause dysfunctions of certain neural circuits that underly their distinct symptoms, making it appropriate to investigate functionality of representative neural circuits. In fact, correspondingly, recent studies suggest that aggregated proteins propagate along specific neural networks, which further suggest importance of neural circuits as disease models for understanding their pathological mechanisms. To date, various neurodegenerative disease models have been established, including the use of cerebral organoids for Alzheimer’s disease (AD) [81, 82], midbrain organoids for Parkinson’s Disease (PD) [83, 84] and motor neuron organoids for Amyotrophic Lateral Sclerosis (ALS) [85, 86].

In addition to investigation on protein aggregation and cellular death, the functionality of NDD organoid models is gradually coming to light. For instance, APP mutant and PSEN1 mutant cerebral organoids were studied as AD models [87]. The authors observed increased synchronous bursts in both of the AD models, mimicking hyperexcitability and hypersynchronization observed in early stages of AD mouse models and patients [88, 89]. Chen et al. proposed an alternative model for pathogenesis of AD through mimicking blood-brain barrier leakage by serum exposure [90]. The exposed organoids showed synaptic loss and reduced network activity as characterized by a decrease in number of spikes, bursts and synchronization. When investigating cerebral organoid network activity in a model of amyotrophic lateral sclerosis/frontotemporal dementia (ALS/FTD), the authors found that the connectivity was comparable between mutant and control organoids despite neuronal loss in mutant organoids, demonstrating neuronal plasticity [91].

A study by Foliaki et al. investigated the electrophysiological properties of cerebral organoid models generated with cells from donors representing Creutzfeldt Jakob disease and PD along with Down syndrome. In early stages (3–4 months), the control and the disease model organoids exhibited no difference in activity. Interestingly, by 6–10 months, the disease model organoids demonstrated slower burst rates and longer periods between network events, as well as neural oscillation changes [92]. Since PD primarily affects dopaminergic neurons, midbrain organoids have been used to study PD. A recent study observed decreased firing rate and number of spikes that was evident following the maturation of PD-model midbrain organoids with SNCA mutation [93]. Studies on the network functionality of NDD *in vitro* are increasing, and further development is expected to uncover pathogenesis mechanisms of NDDs and to serve as platforms for drug development.

Besides the diseases with genetic causes, models have also been developed for diseases caused by damages induced by external triggers including traumatic brain injury, viral infection and malaria [94–97]. Substance abuse is also modeled with brain organoids [98, 99]. Functional analyses of network activity upon drug applications have been demonstrated [100]. Analyses on circuit functions would be useful for a deeper understanding on the chronic and acute effects of the external damages in the models.

### Psychiatric diseases

Psychiatric diseases exhibit diverse symptoms that affect higher-order brain functions. Our understanding of the etiology and pathophysiology underlying these disorders remain limited. With no definite biomarkers or anatomical changes, diagnosis of psychiatric diseases largely relies on clinical symptoms such as behavioral and cognitive impairments. *In vivo* studies of patients revealed neural circuitry alterations that often span multiple brain regions. A deeper understanding of developmental progression and early network changes associated with psychiatric diseases can possibly result in earlier intervention and more treatment options. Due to the complexity of psychiatric diseases, it is extremely difficult to model the disorders *in vitro*. Cellular or molecular analyses have not been very effective to decipher the higher-order dysfunctions and it is now clear that analyses at a network level are necessary to study psychiatric disease. Fortunately, recent progress in technology and cultures has shown promises toward understanding psychiatric diseases *in vitro*.

Three-dimensional brain organoids have opened new opportunities in studying the developmental progression of psychiatric diseases. Several papers reported abnormalities in neurogenesis and neurodevelopment using organoid models of schizophrenia and bipolar disorder, including reduced neural progenitor cell proliferation, disrupted neural differentiation, and altered excitatory-inhibitory balance [101–104].

Recent studies have attempted to explore the functional alterations in organoids modelling psychiatric disorders. Schizophrenia patient-derived organoids and bipolar disorder patient-derived organoids exhibited diminished response to stimuli, although the baseline electrical activity did not differ from the control [105, 106]. These studies suggest that functional study on neural circuits could provide important insights into deregulation mechanisms of neural circuits underlying the disease symptoms, and the emerging field could advance further by developing better models and analyses.

In this last decade, we have seen major advancements in brain organoids and disease models using them. Their complexity has offered unprecedented opportunities in uncovering the functional aspects of neurological diseases at a cellular and network level. More advanced models combined with diverse technologies would provide a deeper understanding of not only the diseases but also our normal brain functions and mechanisms.

## Brain organoid engineering

### Multi-regional organoids

Inter-regional circuit models are crucial in understanding brain functions and mechanisms. Beyond the models of local and regional circuitry, researchers have developed more advanced strategies for modeling neural circuits structured at a relatively larger scale. Importantly, developmental interaction between brain regions is essential to form proper structures of the brain, and the interaction facilitate formation of neural networks. To model the inter-regional interaction, multiple approaches have been proposed with their effectiveness demonstrated.

The first approach is to induce developmental body axes for generating multiple regions within an organoid along the axes. During brain development, the dorsal forebrain produces excitatory neurons and becomes the cerebral cortex. Meanwhile, the ventral forebrain becomes ganglion eminence and produces inhibitory neurons which migrate tangentially to the dorsal forebrain and are incorporated into the cerebral cortex. To reproduce this process *in vitro*, a method of locally inducing ventral differentiation within an organoid has been proposed [107]. By incorporating cells that secrete the ventralization factor, SHH, an organoid gains the dorsoventral axis and forms both dorsal and ventral sides within an organoid. Local expression of SHH has been demonstrated by using light-activatable Cre recombinase system [108]. Toward this purpose, light-activatable HH signaling modulator compounds have been developed [109, 110].The first approach is to induce developmental body axes for generating multiple regions within an organoid along the axes. Interestingly, spontaneous formation of dorsoventral axis within an organoid has been achieved by culturing the organoid on a gel and exposing the organoid to SHH or equivalent compounds [111].

Another type of approach is direct fusion of multiple organoids that models different regions of the brain. Various types of brain organoids are fused to model interaction between different pairs of regions. Such fused organoids are widely recognized as “assembloids” [112–115]. This approach is also effective in modeling the dorsal-ventral interaction of forebrain [112], in which GABAergic inhibitory neurons generated from the ventral region migrate to the dorsal organoids. Furthermore, an assembloid generated by fusing three organoids including the cerebrum, motor nerves, and muscles, has been reported, demonstrating its applicability to model complex neural circuits [116]. The assembloid approach provides a simple and powerful approach to model connections of multiple regions beyond the body axis and regardless of their native distance in the nervous system.

### Construction of the brain organoid circuits

Though the above mentioned approaches have shown feasibility in modeling the interactions between multiple regions and creating circuit structures, none of them generate an axonal tract that appears to be the signature structure in the brain and serves as a critical hub for the inter-regional connections. In the brain, axonal tracts in and between cerebral hemispheres form the white matter. While distal cortical regions are connected through the white matter (extrinsic pathway) in the human brains, the corresponding axons in the mouse brains are either fewer or located inside the grey matter (intrinsic pathway) [117]. This structural difference of the axonal tracts might lead to functional differences of the brains between the species, given that axonal tracts could serve as a structural mechanism to support the massive information-processing and complex coordination in human brains. To understand the significance of this structural difference, and to accurately model the human macroscopic neural circuits, it is essential to create a model circuit in which human brain organoids are connected via axonal tracts.

The first step towards reconstructing a brain circuit that mimics the distal connections of different brain regions is the establishment of an *in vitro* axon tract model that contains self-assembled bundles of axons. With this in mind, we have previously developed a method to fabricate motor nerve organoids in microfluidic devices [118]. In this method, axons extended from the organoid is physically guided by a wall of a culture vessel into a thin microchannel in which axons extend unidirectionally and spontaneously assemble into a bundle through axo-axonal interaction. Based on this technique, a method was established to connect two cerebral organoids with an axon bundle by using the same culture vessel (microdevice) [119]. Human iPS cell-derived cerebral organoids are placed in two small chambers at the both ends of the microchannel. The organoids extend their axons into a microchannel between them, and eventually the axons reciprocally reach the cerebral organoid that has been placed on the other end of the microchannel. The two interconnected cerebral organoids can be obtained as one tissue, mimicking a cortico-cortical connection in the brain. We call these connected organoids “connectoids”. The inter-organoid connection could be also formed by connecting two organoids with reciprocally extending axons within a tube of gel [120, 121].

The difference between assembloids and connectoids lies in the axon bundles which can be useful for understanding how axonal tracts assemble and form. In addition to the importance of the axon bundle in structural and developmental studies, functionality of the tissue could be dramatically changed by the structure. Using multi-electrode array (MEA), the activities of connectoids were observed to be significantly more complex and active than those of single cerebral organoid and cortico-cortical assembloids [122], suggesting importance of macroscopic structure and architecture of neural circuits *in vitro*.

## Perspective: developing functions of neural circuits *in vitro*

### *In vitro* neural circuits at a thrilling intersection of multiple disciplines

**Fig. 3:**
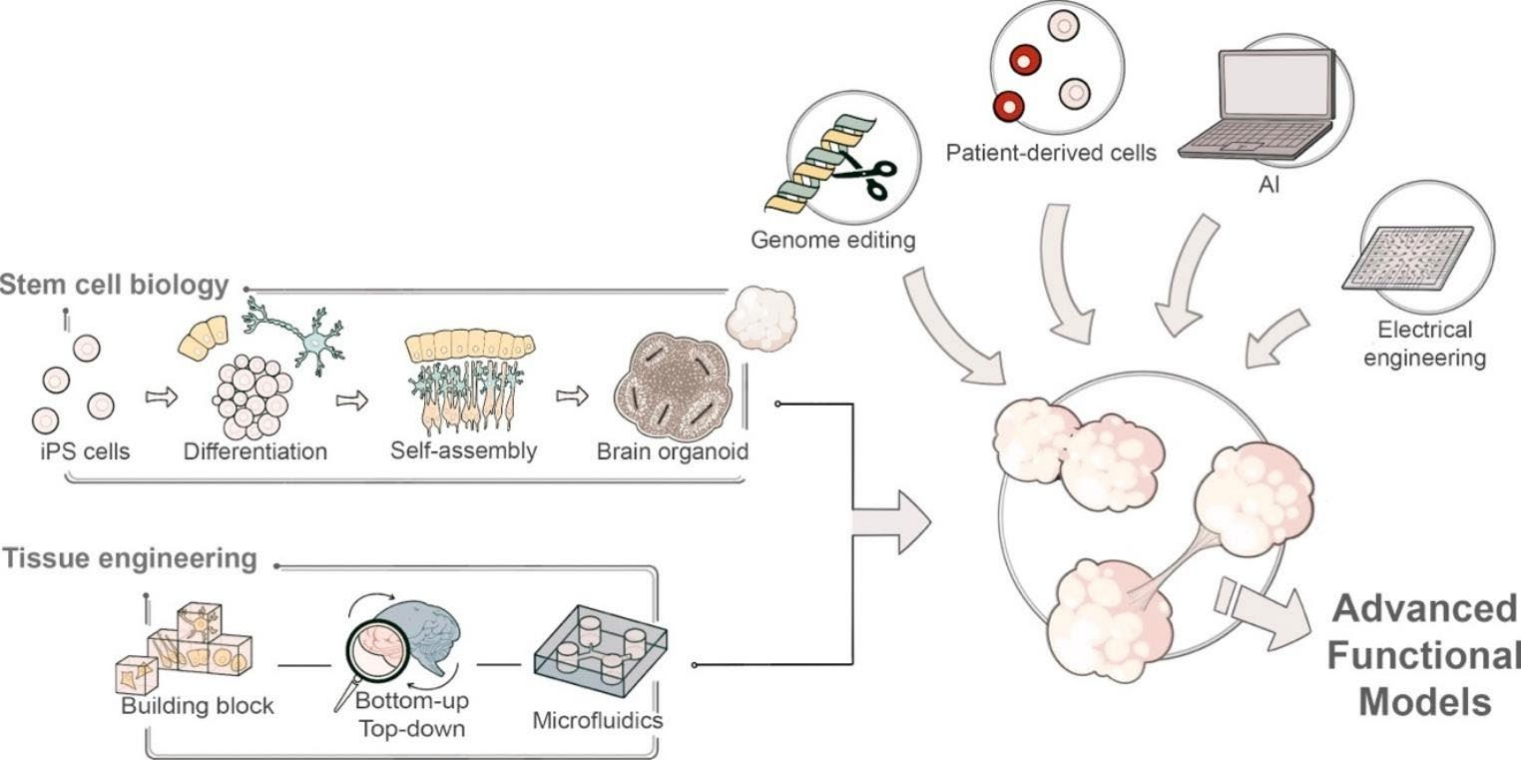
Development of advanced functional *in vitro* neural circuits

As described, organoid models of neural circuits are quickly advancing through the application of bioengineering technologies on biologically generated tissues, in parallel to the advances in two-dimensional neural circuits, where recent improvements largely depend on bioengineering techniques (Fig. 2). The neurons with their elementary functions acquire the ability to exhibit assembly and orchestration of neural activity as a circuit, which recapitulates the mechanistic functions underlying the higher-order functions of the brain (Fig. 1). The examples of bioengineered neural networks highlight the progress of *in vitro* neural cultures as a result of fruitful collaboration between stem cell biology and tissue engineering (Fig. 3). This is truly exciting since the two fields of study share the same ultimate goal to build and learn from organs, although different approaches have been applied. Combining the strength of the two fields certainly boosts a new (yet anticipated) stream of research in neural circuit modeling *in vitro*. Biology-driven approaches for generating neural culture and tissues, which heavily rely on cellular self-organization processes, could in theory generate tissues precisely mimicking the brain as the cells are programmed to generate the whole organ. However, self-assembly often suffers from a lack of environmental context which is supplied in embryos. Obviously making the whole embryo would be ethically unfeasible for the study of neural circuit models. Instead, a dash of engineering techniques could go a long way to supply missing guidance and components to tissues, and thus obtain models of interest at the level of desired complexity and structures. Other technologies also became essential to model *in vitro* neural circuits. To create model tissues in diverse context including diseases, genome editing and other molecular genetic approaches are becoming as important as usage of patient-derived cells. To understand activity of neurons and their functions, it is becoming essential to incorporate other disciplines including artificial intelligence (AI) and electrical engineering, which will be described below. The merging interest and participation from various fields of study on *in vitro* neural circuits create a unique opportunity for the researchers to explore new ways to code and decode neural activity patterns along with developing the circuits at the same time. This could construct a new platform and scientific community to probe fundamental mechanisms of neural circuits underlying higher-order functions of the brain.

Stem cell biology and tissue engineering technologies converge into organoid engineering technologies. By incorporating other technologies including genome editing, patient-derived cells, artificial intelligence, and electrical engineering would generate advanced functional *in vitro* models.

### Structure-function relationship

A demonstration of *in vitro* circuits of three snail neurons that generate oscillation is one of the simplest and most sophisticated examples of *in vitro* network executing clear functions [123]. Since then, advanced methods to create complex and ordered neural circuits have been developed in the hope for the biomimetic circuits to be more functional. Various approaches proved that the structured and organized circuits could have more functional capacity than random circuits in both two-dimensional and three-dimensional conditions.

Random neural circuits *in vitro* often generate simple periodic burst activity, which by itself could be considered as a functional signature of the neural circuits. The periodic bursts could be generated by strong recruitment of individual neurons to fire together with other neurons to form synchronous burst activity, due to their tight and strong synaptic connections and uniformness of the circuits. This could be also considered as a hyperactive (possibly epileptic) pattern in which too many neurons fire together and hence its information coding capability is hindered, which indicate that too strong and too uniform connections limit functionality of neural circuits. Adequate amount of inhibitory neurons and astrocytes are also critical to maintain complex activity patterns of the networks. In contrast to the random network, regional circuits in the brain are connected with other regions with their axons projected between the regions, allowing information to flow from one region to another, and the whole brain to function coordinately. Additionally, the axonal projections could serve as structural constraint for the circuit to protect them from excessive connection and thus epileptic seizures. The axonal connections also could provide time delay as important factor for functionality of computation and oscillation of neural circuits. One could speculate that the structural constraint can provide convergence points for information and neural activity, whereas the cortical regional networks as divergence segments, which could serve as an important principle for information processing. Thus, neural circuits that contains regional circuits that are inter-connected with axons could be useful for assessing the importance of the convergence/divergence structure motif. Due to structural and functional complexity, the connected organoids could be an ideal platform to assess these hypotheses and functional importance of the inter-regional connections and to formulate a fundamental structural requirement for maximizing functionality of neural circuits. Two-dimensional cultures with the macroscopic separation and connections between segmented neural circuits have demonstrated promising structural contribution to their network activity [124, 125]. Once we understand the basic requirement to increase functionality of *in vitro* neural circuits, more unique brain region- or input- specific functions should be modeled by incorporating more organoids in numbers and types into the circuits.

### Unlocking and understanding higher-order functionality of *in vitro* neural circuits

**Fig. 4:**
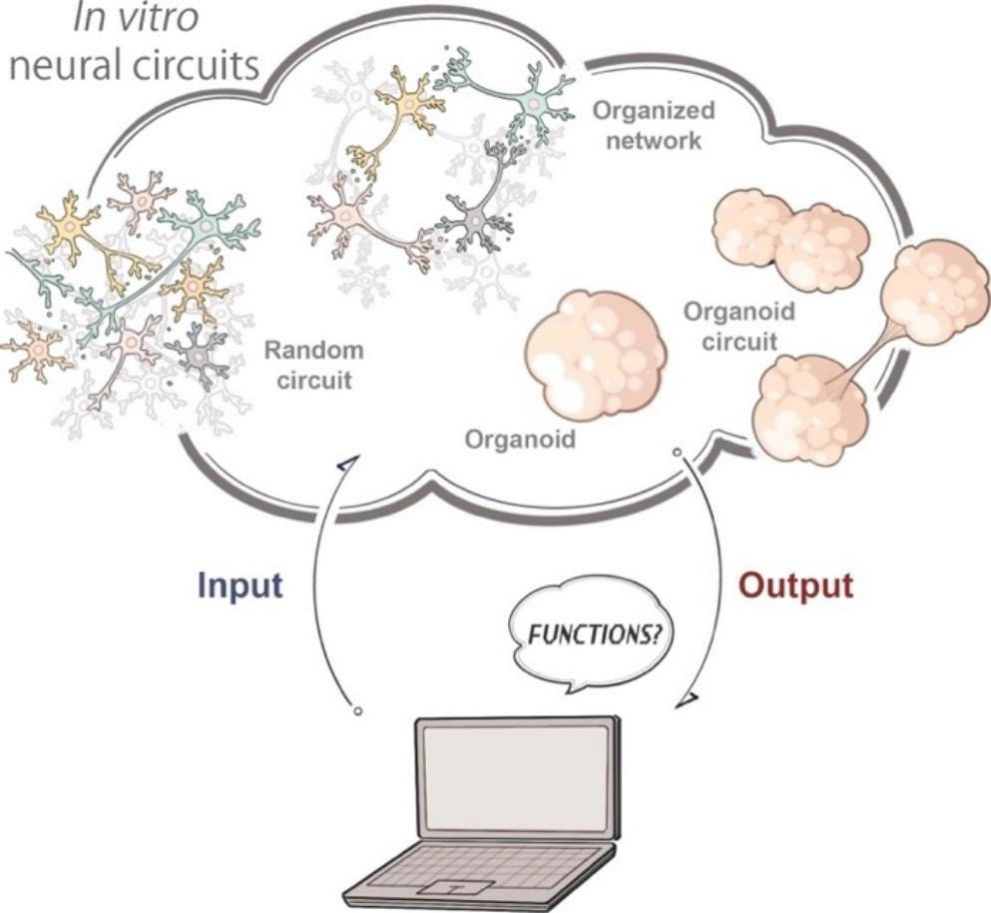
Computer-assisted understanding and generation of higher-order functions of neural circuits

*In vitro* neural circuits have been demonstrated to exhibit functionality including synchronized bursts, oscillation, network propagation, and neural avalanches. These activity patterns generated as neural circuits could be considered as mechanistic functions that provide foundation for higher-order functions of the brain. One of the important questions is whether higher-order functions could emerge in the neural circuits that exhibit mechanistic functionality. This question leads to another question of whether higher-order functionality could be appreciated by observers, even if the neural circuits have the capability of executing higher-order functions in itself. To answer these questions, it is essential to provide a system to relate input to the neural circuits and output from the circuits (Fig. 4). Since most higher-order functions require plasticity of neurons to execute, a closed loop stimulation system would be required to assess these important questions. In this system, neurons receive inputs and generate output signals which would be recorded and instantly processed by computers or electrical components and then it generate the input sequences for the circuits. In a closed loop setup, neural circuits are connected to virtual space where they can interact with the hypothetical external environment. To achieve this, two technological parts make up these closed-loop systems: the interface and the processing. The interface between the biological component (typically, analog signal) and the physical component (typically digital signal) is important to achieve bidirectional communication over analog-digital conversion. These interfaces includes both recording of neuronal activity and stimulation equipment of neural activity [126]. The neuronal recording must be spatiotemporally precise and needs to be captured from a wide range of culture area. Most of the systems are either electrical, in particular by using networks of electrodes (MEA [127, 128]), or optical, in particular by the use of optogenetics and fluorescent activity indicators [129]. The processing part is also an important step for bio-hybrid systems [130, 131]. Indeed, a key feature of these systems is the real-time processing and decoding of neural signals with super low latency (< 1-100 msec) to drive feedback for modulation or replacement of neural function. To this end, enhancing biohybrid systems with artificial neural networks (ANN) is the emerging strategy to achieve complex interactions between biological and artificial neurons [132]. These ANNs can be used for detection and classification but also to reproduce the biological neural network for replacement [125, 132, 133].One approach to achieve this is a neuromorphic system allowing bidirectional communication in real time [134, 135]. Recent studies demonstrated that *in vitro* neural circuits could be incorporated into computational recurrent neural network (RNN) or a “reservoir” to achieve tasks, suggesting that employing the mechanistic structure for artificial intelligence could be a powerful approach for understanding higher-order functionality of neural circuits [136]. Another notable example is that *in vitro* neural circuits have been investigated to achieve responsive game task [137], in which “free energy principle” theory has been examined. They utilized noise to destabilize the network to manipulate the neural circuit activity. With these approaches, researchers demonstrated maneuvers of objects or robots to achieve tasks, suggesting that *in vitro* neural circuits are capable of processing information and executing higher-order functions. These highlights that *in vitro* neural circuits could be ideal for testing theories and methods to unlock the ability of neural circuits and execute higher-order functions. Higher-order functionality of the biological *in vitro* neural circuits are still limited at the current stage but combining closed loop systems with circuit models equipped with more sophisticated structures would pave the way to unleash advanced higher-order functions, and lead to better understanding on the basic principles of neural network mechanisms of the brain. *In vitro* neural circuits could be ideal for both exploring methods to unlock higher-order functions and inventing new methods to effectively process information inspired by the neural circuits.

Do neurons have higher-order functions? We could investigate this by real-time interaction with them through a system with continuous input and output that provides virtual environments.

## Concluding notes

### Current limitations

Human stem cell-derived brain organoids provide numerous advantages to model brain functions and structure. However, currently there are a few major limitations. One of the potential concerns is the variability between organoids. Organoids often differ in their structures and activity patterns even within the same batch of organoids from the same iPS cell line, resulting in difficulty to control their quality. Cell-line-to-cell-line variability also exists. Relatively long time required for maturation and production of some of the cell types could also a limitation. Human brain organoids need a few hundreds of days for their maturation, which is critical for producing some of the late-emerging cell types including upper layer neurons and astrocytes. Also, the brain organoids usually lack major cell types and structures, including those for the blood and immune systems. By resolving these issues, the brain organoids would have better ability to model the human brain.

Considering the functions of brain organoids, one of the most critical limitations of the current organoids is the lack of input and output as stated above. This is critical not only for understanding their hidden functional capability, but also for creating functional circuits within or with the organoids through activity-dependent synapse formation and organization mechanisms. Another limitation is the recording techniques. Although high density electrode arrays and wide-field calcium imaging techniques enables recording of neuronal activity, it is still difficult to record from all neurons in the complex tissue with substantial three-dimensional size.

Engineering techniques are rapidly developing for brain organoids, but there are many technical limitations to overcome. For example, it is currently difficult to build complex three-dimensional structures that are compatible with long-term culture and observation of the organoids. It is also difficult to establish stable long-term gradient of morphogens to establish body axes within organoids. While numerous technical challenges exist, we believe that new technologies in biology and engineering will continue to be invented to advance the construction and modeling of functional neural circuits.

### Conclusions

In this review, we overviewed research progress and discussed perspective of *in vitro* neural circuits and their ability and potential to acquire functions. The field is quickly advancing and changing as exciting discoveries and inventions emerge almost every week. We apologize that many important and interesting studies are not mentioned in this review due to the space limitation. We hope that this review helps the readers to know current advances in the field and to feel the potential for future developments.
